# 5‐Azacytydine and resveratrol reverse senescence and ageing of adipose stem cells via modulation of mitochondrial dynamics and autophagy

**DOI:** 10.1111/jcmm.13914

**Published:** 2018-10-28

**Authors:** Katarzyna Kornicka, Jolanta Szłapka‐Kosarzewska, Agnieszka Śmieszek, Krzysztof Marycz

**Affiliations:** ^1^ Department of Experimental Biology The Faculty of Biology and Animal Science University of Environmental and Life Sciences Wroclaw Wroclaw Poland; ^2^ Faculty of Veterinary Medicine Equine Clinic ‐ Equine Surgery Justus‐Liebig‐University Gießen Germany

**Keywords:** adipose‐derived mesenchymal stem cells, autophagy, metabolic syndrome, mitochondria, mitophagy, stem cells

## Abstract

Obesity and endocrine disorders have become prevalent issues in the field of both human and veterinary medicine. Equine metabolic syndrome is a complex disorder involving alternation in metabolism and chronic systemic inflammation. It has been shown that unfavourable microenvironment of inflamed adipose tissue negatively affects adipose stem cell population (ASC) residing within, markedly limiting their therapeutic potential. ASCs_EMS_ are characterized by increased senescence apoptosis, excessive accumulation of reactive oxygen species (ROS), mitochondria deterioration and “autophagic flux.” The aim of the present study was to evaluate whether treatment of ASCs_EMS_ with a combination of 5‐azacytydine (AZA) and resveratrol (RES) would reverse aged phenotype of these cells. For this reason, we performed the following analyzes: molecular biology (RT‐PCR), microscopic (immunofluorescence, TEM) and flow cytometry (JC‐1, ROS, Ki67). We evaluated the mitochondrial status, dynamics and clearance as well as autophagic pathways. Furthermore, we investigated epigenetic alternations in treated cells by measuring the expression of TET genes and analysis of DNA methylation status. We have demonstrated that AZA/RES treatment of ASCs_EMS_ is able to rejuvenate these cells by modulating mitochondrial dynamics, in particular by promoting mitochondrial fusion over fission. After AZA/RES treatment, ASCs_EMS_ were characterized by increased proliferation rate, decreased apoptosis and senescence and lower ROS accumulation. Our findings offer a novel approach and potential targets for the beneficial effects of AZA/RES in ameliorating stem cell dysfunctions.

## 1 INTRODUCTION

Obesity and endocrine disorders have become common problems in the field of both human and veterinary medicine. It is estimated that approximately 19%‐40% of the horse population is obese^1^ and 22%‐29% suffer from hyperinsulinemia.^2^ Regional adiposity, obesity, hyperinsulinemia, insulin resistance (IR) and laminitis or susceptibility to laminitis are the major characteristics of equine metabolic syndrome (EMS).^3^ It is worth noting that recent data have indicated that obesity should not be used as a diagnostic criterion^4^ EMS is a complex disorder involving alternation in metabolism and chronic systemic inflammation.^5^ However, due to its complexity, understanding EMS pathophysiology is still elusive.

In recent years, many studies have indicated the involvement of oxidative stress in metabolic syndrome (MS). A close link between inflammation and oxidative stress plays a pivotal role in the development of IR. Moreover, reactive oxygen species (ROS) – produced either by mitochondria or in other sites within or outside the cells – damage proteins and organelles and, in consequence, initiate the degenerative process. Thus, strategies to treat accelerated ageing in MS should focus on modulation of mitochondrial dynamics.

Adipose tissue is not only an energy storage, but also a highly active, endocrine organ secreting hormones and cytokines. In our previous study, we have shown increased expression of IL‐6 and TNF‐α in adipose tissue and serum of Welsh ponies suffering from EMS. Similarly, many other authors have described increased levels of pro‐inflammatory cytokines, including IL‐1 β, IL‐6 and TNF‐α in serum and adipose tissue in EMS individuals^6^, ^7^, ^8^ It has been shown that TNF‐α is directly implicated in IR development by interfering with insulin signalling.^5^, ^9^ This adverse microenvironment of adipose tissue negatively affects adipose stem cell population (ASC) residing within it.

Adipose stem cells are considered to be ideal for application in regenerative medicine, as they can differentiate into multiple lineages both in vitro and in vivo. Moreover, ASCs are well known for their immunosuppressive properties. They were proved to secrete a wide range of proteins in extracellular microvesicles (MVs). Robust secretion of trophic factors strongly correlates with clinical outcome in a wide range of applications. In addition, their huge advantage over mesenchymal stem cells (MSCs) isolated from other sources, like bone marrow, is that they are easily harvested and isolated in large quantities with minimal donor‐site morbidity.

Due to their unique properties, ASCs hold promise in treating multiple disorders, including graft versus host disease,^10^ multiple sclerosis,^11^ diabetes mellitus^12^ and autoimmune‐induced diseases.^13^, ^14^ Moreover, their application in bone and cartilage regeneration as well as wound healing was widely investigated.^15^, ^16^, ^17^, ^18^ There has been a growing number of ASC‐related studies demonstrating the ability of these cells to decrease IR, promote regeneration of pancreatic beta cells and suppress autoimmunity in the course of diabetes.^19^

However, our previous data indicated a severe deterioration of ASCs isolated from EMS horses (ASC_EMS_), which questions their therapeutic utility in MS treatment. According to our results, ASCs_EMS_ are characterized by limited proliferation potential, increased senescence, apoptosis, excessive accumulation of ROS and mitochondria deterioration.^3^, ^20^, ^21^ In consequence, “autophagic flux” was observed in those cells – a protective mechanism that helps metabolically impaired cells maintain multipotency and stemness. During chondrogenic and osteogenic differentiations, increased autophagy provides ASC_EMS_ precursors to macromolecules, adenosine triphosphate and amino acid synthesis.^22^, ^23^ Accelerated degradation of heterochromatin associated with the inner nuclear membrane and increased amounts of 5‐methylcytosine in DNA of ASCs_EMS_ also indicate epigenetic alternation in these cells. Moreover, secretion of MVs, significant in regeneration and cellular communication, was markedly reduced in those cells. Increased levels of ROS result in impairment of mitochondrial dynamics and endoplasmic reticulum stress. Elevated mitochondrial fission and decreased mitochondrial membrane potential (MMP) may be particularly responsible for the activation of the apoptotic pathway in those cells. All these phenomena strongly limit the therapeutic potential of ASCs_EMS_ and bring serious consequences for their usefulness in regenerative medicine.

Increasing body of evidence suggests that allogeneic MSCs may elicit an immune response in a recipient animal and that allogeneic donor MSCs are not fully immune‐privileged, as previously claimed. The formation of anti‐allogeneic MSC antibodies, following an intradermal allogeneic MSC injection, was observed in horses.^24^, ^25^ These results highlight the potential risk of allogeneic MSC application.

Given the aforementioned facts, it needs to be considered in EMS‐diagnosed individuals whether autologous graft of ASCs will be therapeutically valuable and effective. Thus, alternative approaches, aiming to rejuvenate autologous ASCs in vitro to enhance their regenerative potential have become the centre of scientific attention.

The approach of ASC_EMS_ rejuvenation presented by our group relies on two distinct features of these cells – ROS accumulation and epigenetic alternation. In our research, we decided to combine two molecules targeting mitochondria and DNA of ASCs_EMS_ in order to reverse their aged phenotype.

Resveratrol (RES) has been shown to exert immunomodulatory, anti‐inflammatory and antioxidative effects.^26^, ^27^ Moreover, RES has gained widespread attention, because of its ability to prolong lifespan and protect against age‐related disorders in different animal models.^28^ A landmark paper by Baur et al^29^ revealed that RES supplementation can reverse diabetic physiology of mice fed a high‐fat diet (HFD), back to that of mice on a standard diet. In addition, it increased insulin sensitivity, activated 5′AMP‐activated protein kinase (AMPK)/Peroxisome proliferator‐activated receptor gamma coactivator (PGC)‐1α signalling, improved mitochondrial parameters, reduced insulin‐like growth factor 1 levels and enhanced motor and liver function of HFD mice. In mammalian cells, RES activates SIRT‐1, which results in an improvement of cellular function and health of the organism. Mitochondrial oxidative stress is a frequently observed phenomenon in diabetes and MS, causing ROS‐dependent accelerated ageing. Lagouge et al^30^ has demonstrated that RES improves mitochondrial function and protects against metabolic disease by inducing PGC‐1α and SIRT1 activity. Our own data^31^ revealed that polyurethane/polylactide‐based material doped with RES decreased senescence and oxidative stress of ASCs. The fabricated material directed ASCs towards an osteoblast‐like phenotype, which indicated its potential application in regenerative medicine.

On the other hand, 5‐azacytidine (AZA) is easily incorporated into DNA and inhibits methylation pattern of specific gene regions.^32^ It was shown that AZA treatment of hepatocyte‐like cells resulted in an enhancement of metabolic and enzymatic activities of these cells.^33^ A study conducted by Yan et al^34^ revealed that AZA improved osteogenic differentiation potential of human aged ASCs. Our recent research confirmed beneficial effects of AZA, as it reversed aged phenotype of ASCs by decreasing apoptosis and enhancing proliferation rate.^35^

Taking into consideration biological role of micro RNAs (miR) in modulation of cell proliferation and senescence, in presented study we investigated the expression of miR‐24 and miR‐519d. MiR‐24 plays important role in inflammation, cell migration and many diseases. Moreover, it was shown that oxidative stress leads to up‐regulation of miR‐24 an in consequence to apoptosis.^36^ On the other hand, miR‐519 was shown to block autophagy.^37^ It exerts its role by targeting Beclin‐1, ATG10, and ATG16L1.

The aim of this study was to investigate whether the treatment of ASCs_EMS_ with a combination of AZA/RES would reverse aged phenotype of these cells. In order to investigate the effectiveness of ASC_EMS_ rejuvenation, we performed both molecular biology and microscopic analyzes. We evaluated mitochondrial status, dynamics and clearance as well as autophagic pathways. Furthermore, we investigated epigenetic alternations in treated cells and evaluated whether AZA/RES treatment influenced surface antigen expression.

## 2 MATERIALS AND METHODS

All reagents and chemicals used in this research were purchased from Sigma‐Aldrich (Poznań, Poland), unless indicated otherwise.

### 2.1 Research material preparation

#### 2.1.1 Experimental animals

The study involved thirty, mixed sex, age – matched (8‐12 years) horses, classified into the EMS group – consisted of animals suffering from EMS (n = 15) and the control group of healthy individuals (n = 15). Qualification to the experimental groups was performed based on detailed interviews with owners, body weight, body condition score, cresty neck score, existing laminitis, resting insulin levels, blood glucose levels, combined glucose – insulin test and leptin concentration. Comprehensive characteristic of the animals used in this study is presented in Table 1.

**Table 1:** Criteria for dividing the horses into the experimental and control groups

Group	Age	Baseline serum insulin (mIU/mL)	Insulin (mIU/mL) 60 minutes post oral sugar administration	Baseline glucose (mg/dL)	Glucose 60 minutes post oral sugar administration (mg/dL)	BCS	CNS	LEP (ng/mL)	Wt (kg)
EMS	12 ± 2	180 ± 20	260 ± 11	72 ± 11	163 ± 26	7.2 ± 0.2	3.9 ± 0.7	4,8 ± 1,2	680 ± 30
Control	11 ± 2	13 ± 4	36 ± 3	84 ± 7	88 ± 5	6.4 ± 0.3	1.9 ± 0.6	1,1 ± 0,6	590 ± 20

#### 2.1.2 Adipose tissue collection and ASC isolation

White, subcutaneous adipose tissue samples were collected from the horse tail base following the ethical rules and standard surgical procedures as presented elsewhere. The tissue fragments were placed in a sterile Hanks’ balanced salt solution (HBSS) containing 1% of penicillin/streptomycin/amphotericin B (PSA) solution. In order to isolate ASCs harvested material was fragmented by mechanical mincing and digested enzymatically with collagenase type I in a concentration of 1 mg/mL for 40 minutes at 37°C. Obtained homogenate was centrifuged at 1200 *g* for 10 minutes at room temperature. Cell pellet was extensively washed by centrifuging with HBSS (300 *g*, 4 minutes), resuspended in the culture medium and transferred to a T–25 culture flasks. In order to perform the experiments, cells were passaged three times using trypsin solution (TrypLETM; Life Technologies, Carlsbad, CA, USA).

#### 2.1.3 Cell culture

Isolated cells were cultured in Dulbecco's Modified Eagle's Medium (DMEM) containing 4500 mg/L glucose supplemented with 10% of foetal bovine serum (FBS) and 1% of PSA solution. The media were changed every 2 days. The cultures of ASCs were maintained in the incubator with 5% CO_2_ and 95% humidity at 37°C.

#### 2.1.4 Phenotypic characterization and multipotency assay

Mesenchymal character of isolated cells was confirmed by investigating the presence or absence of the following surface markers: CD44, CD45 and CD90 using Becton Dickinson FACS Calibur Flow Cytometer. For analysis, cells were treated with TrypLETM Express solution, rinsed with HBSS and resuspended at total of 5 × 105 cells/mL. Then, specific antibodies (anti‐CD45; Novus Biologicals, Littleton, CO, USA, NB1006590APC, anti‐CD44; R&D Systems, Minneapolis, MN, USA, MAB5449, anti‐CD90, ab225; Abcam, Cambridge, UK) were added to the cell suspension and incubated at 4°C for 20 minutes. The obtained results were analyzed using CellQuest Pro Software (Becton Dickinson, Franklin Lanes, NJ, USA).

Osteogenic, chondrogenic and adipogenic differentiation of the cells were induced using commercial kits (STEMPRO Osteogenesis Differentiation Kit and STEMPRO Adipogenesis Differentiation Kit; Life Technologies). In order to perform the assay, cells were seeded in 24–well plates at a concentration of 2 × 104. Osteogenesis and chondrogenesis were induced during 21 – day period, while stimulation toward adipocytes lasted for 14 days. Cultures expanded in standard growth medium were used as a control group. To evaluate the effects of multilineage differentiation specific cell stainings were performed. Extracellular matrix mineralization was detected with Alizarin Red dye, Oil Red O was apply to detect the intracellular lipid droplets and the formation of proteoglycans was confirmed by Safranin O. The results obtained during staining procedure were analyzed using Axio Observer A1 inverted microscope (Zeiss, Oberkochen, Germany), while the photographic documentation was made using Canon PowerShot digital camera (Ota, Tokio, Japan).

### 2.2 Experimental phase

#### 2.2.1 Cell pretreatment with RES and 5‐azacitidine

After third passage, ASCs were seeded onto 24–well plates at the density of 2 × 104 per well. When cells had been attached, regular culture medium (DMEM containing 4500 mg/L glucose supplemented with 10% of FBS and 1% of PSA) has been changed for medium supplemented with various concentrations of 5‐azacytydine (AZA) and RES. ASCEMS I were cultured in medium containing 0.5 μM of AZA and 0.05 μM of RES while ASCEMS II in 0.5 μM of AZA and 5 μM of RES. Pretreatment lasted for 24 hours, then the experimental medium was replaced by regular culture medium once again. ASCs propagated in DMEM containing 4500 mg/L of glucose supplemented with 10% FBS and 1% antibiotics were used as a control for the experiment. Further experimental procedures performed and described in this study involved four different groups of cells: ASCs isolated from healthy horses (ASCCTRL), ASCs obtained from individuals suffering from metabolic syndrome (ASCEMS) and two groups of ASCs pretreated with different concentrations of RES and 5‐azacytydine‐ ASCEMS I and ASCEMS II.

#### 2.2.2 Cell proliferation assay

All experimental procedures included in proliferation assay were performed after 24 hours of cells propagation. The viability of the cells was determined using resazurin – based dye (TOX8 In Vitro Toxicology Assay Kit, Sigma Aldrich, Poznan, Poland). To perform the experiment, culture media were removed and replaced with 350 μL of dye solution in DMEM/F–12 supplemented with 10% FBS and 1% PSA. Then, cells were incubated at 37°C for 2 hours. The supernatants were collected and transferred into the 96‐well microplate reader (Spectrostar Nano; BMG Labtech, Ortenberg, Germany). Reduction of the dye was measured spectrophotometrically at a wavelength of 600 nm for resazurin and 690 nm as a reference wavelength. In order to estimate the clonogenic potential of cells, they were seeded in a 6‐well plate at an initial density of 1 × 102 as described elsewhere.^38^ After 7 days in culture, biological material was fixed with 4% ice – cold paraformaldehyde (PFA) and stained with pararosaniline. Colonies consisting of more than 50 cells were counted and the efficiency of colony forming (CFU) was calculated using the formula presented below:

$$\begin{array}{cc} & \text{CFU‐fs}(\%)=(\text{number of colonies}>50\text{cells})/(\text{initial cell number})\\ & \times 100\% \end{array}$$

#### 2.2.3 ASCs morphology and ultrastructure

Adipose stem cells morphology was evaluated under epifluorescent microscope (Axio Observer A1; Zeiss), confocal microscope (Cell Observer; Zeiss), electron microscope (SEM, ZeissEvoLS15) and focused ion beam (FIB, Zeiss, Cobra, Auriga 60) microscope after 24 hours of culture. The preparation of biological material for fluorescent microscopy included the following steps: triple washing with HBSS; cells fixation in 4% PFA for 20 minutes; repeated rinsing with HBSS; cell membranes permeabilization with 0.1% Triton X‐100 for 15 minutes at room temperature; cells washing; staining with atto‐488‐labeled phalloidin (1:800) for 30 minutes; counterstaining using Hoechst33258 (1:1000) for 5 minutes. Additionally, MitoRed fluorescent dye was used to stain mitochondria. The staining procedures were performed following the manufacturer's instructions. All photographic documentation was made using Canon PowerShot digital camera.

For SEM analysis, cell cultures were fixed with 2.5% glutaraldehyde for 1 hour at room temperature, dehydrated in a graded ethanol – water mixtures, dried with air for 30 minutes and coated with gold (ScanCoat 6, Oxford).^39^, ^40^ Prepared samples were observed using an SE1 detector, at 10 kV filament tension To perform TEM analysis, the samples were collected and fixed with 2.5% glutaraldehyde for 24 hours at 4°C and proceed as described elsewhere.^22^ Briefly, cells were incubated for 2 hours with 1% osmium tetroxide and counterstained with lead citrate and uranyl acetate. Next specimens were dehydrated in a graded series of acetone, embedded (Agar Scientific Ltd, Essex, UK) and sectioned into ultrathin slices (70 nm). The observations were carried out using FE‐STEM Auriga60 at 20 kV filament tension. Evaluations of mitochondrial morphology and autophagosomes formation were accomplished by taking TEM micrographs from randomly selected areas of ASC. Additionally percentage of abnormal mitochondria (membrane raptures, vacuoles formation) in cells was calculated.

Prior to the analysis of LAMP2 and DNMT1‐ localization, cells were fixed in 4% PFA for 30 minutes and washed three times with HBSS. Whole procedure was conducted based on the protocol presented previously.^22^ Briefly, cells’ membranes were permeabilized with 0.5% Triton X‐100 for 20 minutes at room temperature while unspecific binding sites were blocked with blocking buffer (10% Goat Serum, 0.2% Tween‐20 in HBSS) for 45 minutes. Cells were then incubated overnight at 4°C with primary antibodies against LAMP2 (Abcam) or DNMT‐1 (Abcam), diluted 1:500 in HBSS containing 10% Goat Serum. Cells were then washed again and incubated for 1 hour with goat anti‐mouse secondary antibodies conjugated with atto‐488 (dilution 1:1000; Abcam), avoiding direct light. Subsequently, nuclei were counterstained by incubation with Hoechst33258 for 5 minutes. Cells were observed and photographed using confocal microscope (Observer Z1 Confocal Spinning Disc V.2 Zeiss with live imaging chamber) and analyzed using ImageJ software (Bethesda, MD, USA).

#### 2.2.4 Flow cytometric analysis

All flow cytometry analysis were performed after 24 hours of the experiment. To evaluate the expression of LAMP – 2 and 5‐mathylocytosine (5‐mC), ASCs were centrifuged at 350 *g* for 5 minutes and fixed with 4% ice – cold PFA. The cells were washed extensively with HBSS and incubated with 0.1% Tween diluted in HBSS for 20 minutes. Biological material was incubated with anti‐LAMP2 (ab25631; Abcam) antibody (1:200) or anti‐ 5mC antibody (ab73938; Abcam) solution supplemented with 10% goat serum for 30 minutes at 22°C. Afterwards, the cells were incubated with Alexa 488 goat anti–mouse secondary antibodies (1:500, Alexa Fluor 488; Abcam) for 30 minutes at 22°C.

To assess MMP, the cell pellet were treated with 1 mM JC‐1 reagent (Life Technologies), whereas intracellular ROS were detected using H2DCF‐DA dye in accordance to manufacturer’ instruction. To perform cell cycle analysis, samples were treated with FxCycle PI/RNase Staining Solution in accordance to manufacturer’ protocol. All analytical procedures were conducted with FACS Calibur Flow Cytometer. The results of JC‐1, H2DCF‐DA, 5‐mC, LAMP‐2 and propidium iodide staining methods were analyzed with CellQuest Pro Software (Franklin Lakes, NJ, USA).

#### 2.2.5 Oxidative stress factors and senescence

Oxidative stress and apoptosis were assessed after 24 hours of culture. Supernatants were collected from cultures and subjected to spectrophotometric analysis. Superoxide dismutase (SOD) activity was detected using SOD assay kit, nitric oxide concentration was assessed with the Griess reagent kit (Life Technologies) in accordance to manufacturer’ protocols.

Cellular senescence in ASCs was determined using Senescence Cells Histochemical Staining Kit based on β‐galactosidase activity following manufacturer’ instruction. Furthermore, the number of viable and dead cells were evaluated with the Cellstain Double Staining Kit (Sigma Aldrich). Viable cells nuclei were stained green with Calcein‐AM, whereas dead cells were dyed orange with propidium iodide. All the procedures were performed according to the manufacturers’ protocols. Moreover, staining results were quantified using representative photographs by calculating the percentage of dead and β‐galactosidase positive cells in cultures.

#### 2.2.6 Analysis of gene expression: real‐time reverse transcription polymerase chain reaction

After 24 hours of culture, adherent cells were detached from culture plates, extensively washed with HBSS and homogenized with 1 mL of TRI ReagentTM. Total RNA was isolated according to a phenol – chloroform method described by Chomczynski and Sacchi.^41^ The obtained RNA was diluted in DEPC – treated water. The quantity and quality of received genetic material was estimated using a nanospectrophotometer (WPA Biowave II). Thereafter, enzymatic digestion of genomic DNA (gDNA) following with complementary DNA (cDNA) synthesis were performed using Takara PrimeScriptTM RT Reagent Kit with gDNA Eraser (Perfect Real Time). Each reaction contained 150 ng of total RNA. Both procedures were carried out following the manufacturer's protocol using T100 Thermal Cycler (Bio‐Rad, Hercules, CA, USA).

The quantitative real‐time reverse transcription polymerase chain reaction (qRT‐PCR) reactions were performed using SensiFast SYBR & Fluorescein Kit (Bioline, London, UK) and a CFX ConnectTM Real‐Time PCR Detection System (Bio‐Rad) Each reaction mixture contained 2 μL of cDNA in a total volume of 20 μL, while the primers concentration was 0.5 μM per sample. Sequences of the primers used in the amplification are listed in Table 2.

**Table 2:** Sequences of primers used in qPCR

Gene	Primer	Sequence 5′‐3′	Amplicon length (bp)
LC3	F:	TTACTGCTTTGCTCTGCCAC	213
	R:	AGCTGCTTCTCCCCCTTGT	
Beclin	F:	GATGCGTTATGCCCAGATGC	147
	R:	ATCCAGCGAACACTCTTGGG	
LAMP2	F:	GCACCCCTGGGAAGTTCTTA	139
	R:	TTCGAGGATCTGTGCCAATCA	
GAPDH	F:	GATGCCCCAATGTTTGTGA	250
	R:	AAGCAGGGATGATGTTCTGG	
CHOP	F:	AGCCAAAATCAGAGCCGGAA	272
	R:	GGGGTCAAGAGTGGTGAAGG	
PERK	F:	GTGACTGCAATGGACCAGGA	283
	R:	TCACGTGCTCACGAGGATATT	
PINK	F:	GCACAATGAGCCAGGAGCTA	298
	R:	GGGGTATTCACGCGAAGGTA	
PARKIN	F:	TCCCAGTGGAGGTCGATTCT	218
	R:	CCCTCCAGGTGTGTTCGTTT	
FIS	F:	GGTGCGAAGCAAGTACAACG	118
	R:	GTTGCCCACAGCCAGATAGA	
MFN	F:	AAGTGGCATTTTTCGGCAGG	217
	R:	TCCATATGAAGGGCATGGGC	
p53	F:	TACTCCCCTGCCCTCAACAA	252
	R:	AGGAATCAGGGCCTTGAGGA	
p21	F:	GAAGAGAAACCCCCAGCTCC	241
	R:	TGACTGCATCAAACCCCACA	
Cas‐9	F:	TCCTACTCCACCTTCCCAGG	150
	R:	CTCCGAAACAGCGTGAGCTA	
p62 (SQSTM)	F:	CATCGGAGGATCCCAGTGTG	207
	R:	CCGGTTTGTTAGGGTCGGAA	
IR	F:	CCGTTTGAGTCTGAGGGGTC	254
	R:	ACCGTCACATTCCCGACATC	
TET 2	F:	ATCCTGATCCTGGTGTGGGA	143
	R:	CCTTGACAGGCACAGGTTCT	
TET 3	F:	CAGCCTGCATGGACTTCTGT	188
	R:	GTTCTCCTCACTGCCGAACT	
DNMT‐1	F:	GGCGAAAGCGGACAATTCTG	90
	R:	AGCGGTCTAGCAACTGGTTC	
Mief1	F:	ATGCTGGGCATCGCTACAC	284
	R:	CGGAGCCGTGACTTCTTCAA	
Mief2	F:	AGAACTCTGCCATGGTCTTCT	108
	R:	CGTTCTATTATCAGGCAGGTCC	

Sequences and amplicon length of the primer sets. LC3: microtubule associated protein 1 light chain 3 beta (MAP1LC3B); Beclin: beclin 1, autophagy related (BECN1); LAMP2: lysosomal‐associated membrane protein 2; GADPH: glyceraldehyde‐3‐phosphate dehydrogenase; CHOP: DNA damage inducible transcript 3; PERK: PRKR‐like endoplasmic reticulum kinase; PINK: PTEN‐induced putative kinase 1 (PINK1); PARKIN: parkin RBR E3 ubiquitin protein ligase (PARK2); FIS: mitochondrial fission 1 molecule; MFN1: mitofusin 1; p53: tumor suppressor p53; p21: cyclin‐dependent kinase inhibitor 1A, Cas‐9: caspase‐9; p62: Sequestosome‐1; IR: insulin receptor; TET 2: Tet methylcytosine dioxygenase 2; TET 3: Tet methylcytosine dioxygenase 3; DNMT‐1: DNA (cytosine‐5)‐methyltransferase 1; Mief1: mitochondrial dynamics protein MID51; Mief2: mitochondrial dynamics protein MID49.

To determine miRNA expression, 500 ng of RNA was reverse‐transcribed using a Mir‐X miRNA First‐Strand Synthesis Kit (Takara Bio Europe) and then subjected for qPCR (final volume 20 μL) with SYBR Advantage qPCR Premix (Takara Bio, Kusatsu, Prefektura Shiga, Japonia). The reaction included the initial denaturation at 95°C for 10 seconds, followed by 55 cycles of 95°C for 5 seconds and annealing temperature 60°C for 20 seconds with a single fluorescence measurement (Table 3).

**Table 3:** Sequences of primers used in miRNA expression analysis

Primer miRNAs	Sequence 5′‐3′
miR‐24‐3p	TACCACAGGGTAGAACCACGGA
miR‐159d‐5p	CCTCCAAAGGGAAGCGCTTTCTG

The average fold change in the gene expression of experimental cultures was compared with control cultures and calculated by the 2−DDCt method in relation to the housekeeping gene—GAPDH and U6snRNA for miRNA quantification.

#### 2.2.7 Western blotting

Cells were detached from culture dishes and homogenized in RIPA buffer plus protease inhibitor cocktail. The lysates were centrifuged at 4°C for 20 minutes (14 000 *g*) and supernatants were transferred to new tubes. Thirty micrograms of protein were used for each sample. SDS‐PAGE was performed at 100 V for 90 minutes in Tris/glycine/SDS buffer. Proteins were transferred onto a polyvinylidene difluoride membrane (Bio‐Rad) using a transfer apparatus at 100 V for 1 hour at 4°C in Tris/glycine buffer. After transfer, the membrane was washed with Tris/NaCl/Tween buffer (TBST) and blocked overnight at 4°C with 5% non‐fat milk in TBST. Next, the membrane was washed with TBST and incubated overnight with primary antibody for: mitofusin 1 (MFN) (orb11040; Biorbyt, Cambridge, UK), PINK (orb331233; Biorbyt), caspase‐3 (437800; Life Technologies), beta‐actin (A5441; Sigma‐Aldrich) and mitochondrial fision factor ‐MFF (orb325479; Biorbyt) at a dilution of 1:500. After washing the membrane, solution of appropriate secondary antibody conjugated with HRP was applied. After 2 hours incubation, the membrane was washed again with TBST and incubated with Luminata Forte substrate (Merck, Darmstadt, Germany) and visualized using chemiluminescence method with ChemidocMP (Bio‐Rad).

#### 2.2.8 Enzyme‐linked immunosorbent assay

The total concentration of proteins in cell’ homogenates was determined with enzyme‐linked immunosorbent assay (ELISA) for p53 (My Biosource, San Diego, CA, USA). Assay was performed in accordance with the manufacturer's protocol. Spectrophotometric determination was performed with Epoch BioTek^®^ (Winooski, VT, USA).

#### 2.2.9 Statistical analysis

All experiments were performed at least in three replicates. Differences between experimental groups was estimated using the one‐way ANOVA with Tukey's test. Statistical analysis was conducted with GraphPad Prism 5 Software (La Jolla, CA, USA). Differences with probability of *P* < 0.05 were considered significant. Statistical significance indicated as asterisk (*) when comparing the result to ASCCTRL, and as hashtag (#) when comparing to ASC_EMS_.

## 3 RESULTS

### 3.1 Identification of ASCs characteristics

**Figure 1:**
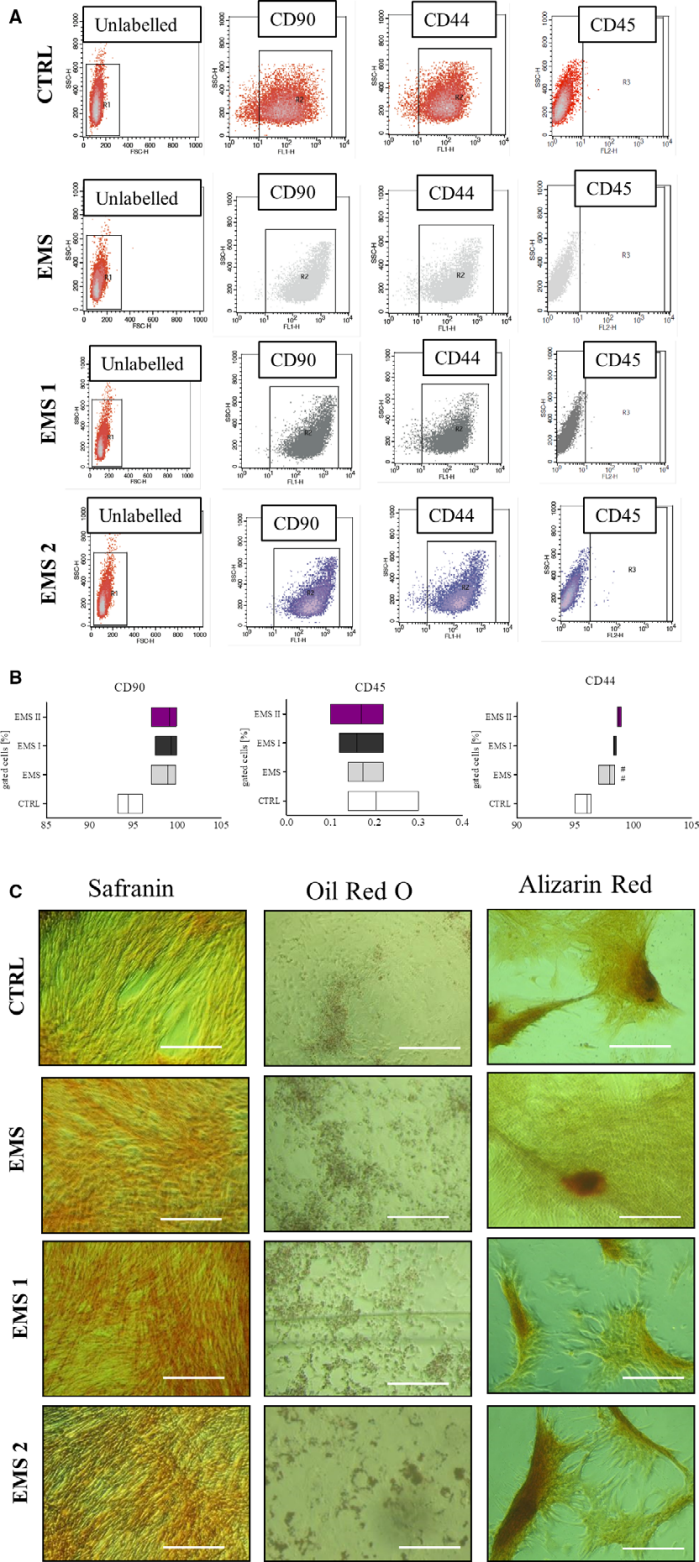
Immunophenotyping and multipotency assay. Representative dot plots from flow cytometry analysis (A). Expression of surface antigens were investigated in cells cultured in control and AZA/RES supplemented medium. Isolated cells were characterized by the expression of CD90 and CD44 while lacked the expression of CD45 hematopoietic marker (B). Multipotency of isolated cells was confirmed by three lineage differentiation (C). In order to confirm formation of proteoglycans during chondrogenesis cells were stained with Safranin O. Intracellular lipid droplets were visualized by Oil Red O while mineralized matrix formed during osteogenesis with Alizarin Red. Magnification ×100, scale bars: 250 μm. Results expressed as mean ± SD. Statistical significance indicated as asterisk (*) when comparing the result to ASC_CTRL_, and as hashtag (#) when comparing to ASC_EMS_. ###*P* < 0.001

Flow cytometer was used to assess immunophenotype of ASCs. Isolated cells were characterized by the expression of CD90 and CD44 surface antigens whereas lack the expression of CD45 hematopoietic marker. Treatment of cells with AZA/RES at two distinct concentration did not affect surface antigens profile (Figure 1A). Expression of CD44 was significantly increased in ASC_EMS_ group (Figure 1B). In order to confirm multipotency of isolated cells, they were differentiated into: chondrogenic, osteogenic and adipogenic lineage. The efficiency of differentiation process was establish by the means of specific staining (Figure 1C). Alizarin Red was applied to visualize mineralized matrix while Oil Red to identify intracellular lipid droplets. Extracellular matrix enriched with proteoglycans was stained with Safranin in order to confirm chondrogenic differentiation.

### 3.2 Growth kinetic and morphology

**Figure 2:**
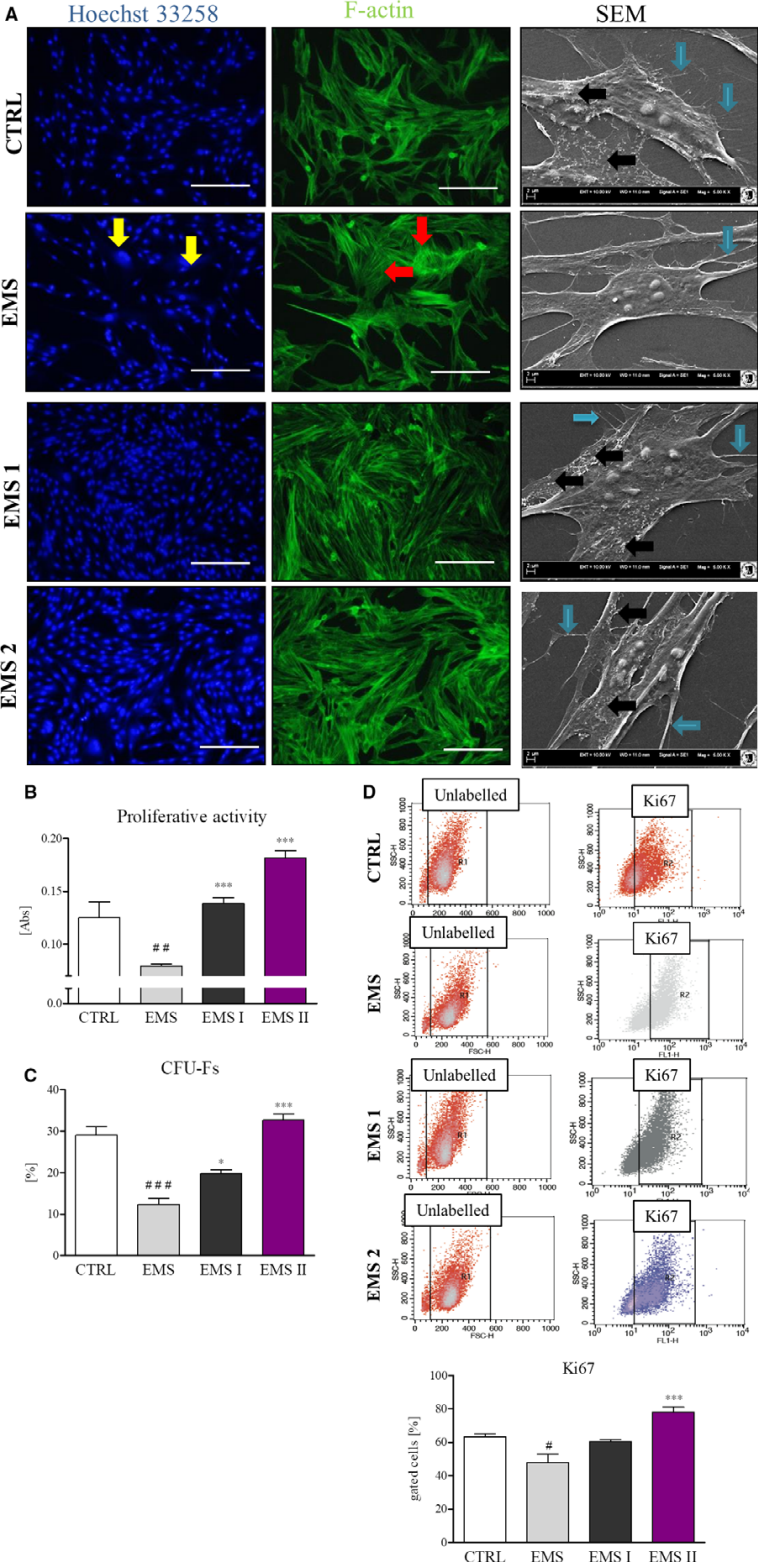
Growth kinetic and morphology of cells cultured in control and AZA/RES (at two different concentrations‐ ASC_EMS__I_ and ASC_EMS__II_ respectively) conditions. In order to perform the assays, cells were cultured for 24 hours and subjected for further analysis. Staining for nuclei (Hoechst 33258) and f‐actin (phalloidin) revealed that some cells in ASC_EMS_ were characterized by enlarged nuclei (yellow arrows, A) and flat, spread‐out cell body with visible stress fibers (red arrows, A). However, treating those cells with AZA/RES diminished occurrence of senescent cells. SEM analysis revealed that control cells (ASC_CTRL_) secrete robust number of microvesicles (indicated with black arrows, A) and displayed formation long cytoskeletal projection called filopodia (indicated with blue arrows, A). On the contrary, secretion of MVs by ASC_EMS_ was compromised. Noteworthy, after culturing those cells in the presence of AZA/RES increased secretion of MVs and formation of filopodia was noted. Using resazurin‐based assay, proliferation of cells was established after 24 hours of culture (B). CFU‐E assay showing percentage of colonies consisting of more than 50 cells between groups (C). Proliferation of cells was also established with flow cytometer and anti‐Ki67 staining (D). Obtained results confirmed, that AZA/RES treatment markedly induce proliferation of ASC_EMS_. Magnification ×100, scale bars: 250 μm. Results expressed as mean ± SD. Statistical significance indicated as asterisk (*) when comparing the result to ASC_CTRL_, and as hashtag (#) when comparing to ASC_EMS_. #,*P < 0.05; ##*P* < 0.01; ###, ***P < 0.001

Proliferation as well as morphology of cells were evaluated after 24 hours of culture in control or AZA/RES supplemented medium. In order to visualize nuclei, cells were stained with Hoechst 33258 whereas actin filaments were stained with phalloidin (Figure 2A). ASC_CTRL_ were characterized by uniform, bipolar, elongated, fibroblast‐like shape, whereas some cells in ASC_EMS_ possesses enlarged nuclei and flat, spread‐out irregular shape. After treatment with AZA/RES, cells in both groups ‐ ASC_EMS I_ and ASC_EMS II_, formed dense monolayer of cells, closely adhering to each other. No enlarged nuclei were noted. Using SEM microscope secretion of MVs and morphology of cells were visualized. Treatment with AZA/RES, resulted in enhanced secretion of MVs in experimental groups comparably to that observed in ASC_CTRL_. Similarly, it enhanced formation of cytoskeletal projections (filopodia) in experimental groups. Proliferation rate was established using resazurin based assay (TOX‐8) in accordance to manufacturer's instructions. Cells were seeded onto 24‐well plates at the initial number 2 × 10^3^ cells/well. Cells isolated from healthy individuals proliferated at significantly higher rate in comparison to ASC_EMS_ (Figure 2B, *P* < 0.01). However, cells treated with AZA/RES displayed significantly enhanced growth rate in comparison to ASC_EMS_ (Figure 1B, *P* < 0.001). Similar trend was observed in the CFU‐E assay as cells in experimental groups formed colonies originated from one cells more frequently in comparison to control and EMS group (Figure 2C, *P* < 0.05 and *P* < 0.001).

Number of actively proliferating cells was further identified and quantified by flow cytometer using anti‐Ki67 antibody. Obtained results indicated on decreased antigen accumulation in ASC_EMS_ (Figure 2D, *P* < 0.05). However, ASC_EMS II_, were characterized by markedly increased number of Ki67 positive cells (Figure 2D, *P* < 0.001).

### 3.3 AZA/RES alleviated apoptosis and senescence in ASC_EMS_

**Figure 3:**
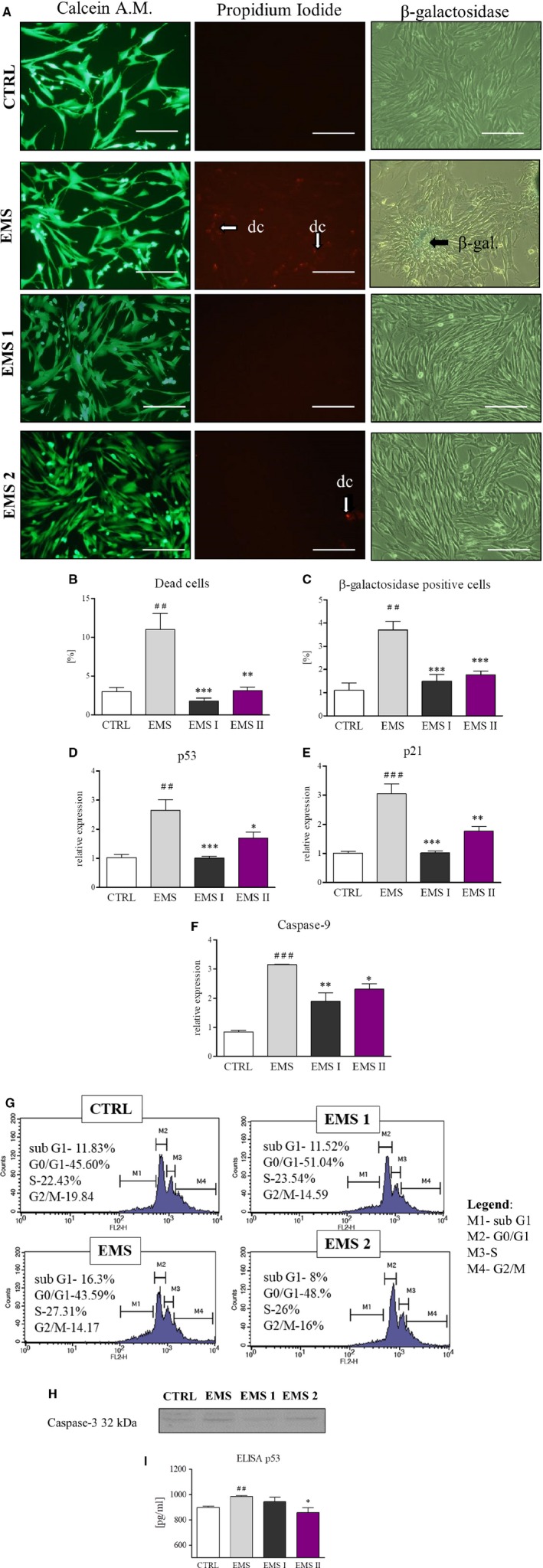
AZA/RES reversed aging and reduced apoptosis in ASC_EMS_. In order to evaluate apoptosis in culture cells were cultured for 24 hours in control or experimental (AZA/RES) condition. Next, cells were subjected to staining procedures and RT‐PCR analysis. In order to visualize live and dead cells in culture Calcein A.M and propidium iodide staining was applied (A). Moreover, senescent cells in cultures were visualized by β‐galactosidase staining (A). Furthermore, data obtained from representative photographs was quantified (B and C). Presented results displayed that AZA/RES treatment markedly reduced number of dead and senescent cells. Apoptosis incidence was also investigated with RT‐PCR for p53 (D), p21 (E), and Caspase‐9 (F). Representative graphs from cell cycle analysis (G). Western blot for caspase‐3 (H) and ELISA for p53 (I), Magnification ×100, scale bars: 250 μm. Results expressed as mean ± SD. Statistical significance indicated as asterisk (*) when comparing the result to ASC_CTRL_, and as hashtag (#) when comparing to ASC_EMS_. *P < 0.05; ##, **P < 0.01; ###, ***P < 0.001

In order to evaluate apoptosis rate in cultures, cells were seeded onto 24‐plates at the initial density 2 × 10^4^ and propagated in control and AZA/RES supplemented medium. Next cells underwent fluorescence stainings and RT‐PCR analysis. Representative photographs are showing the results of live cells staining (calcein), dead cells (propidium iodide) and senescent cells (β‐galactosidase) (Figure 3A). Obtained from the live‐dead staining results were quantified and showed on the graph (Figure 3B). ASC_EMS_ were characterized by significantly increased number of dead cells in comparison to control group (*P* < 0.01), however AZA/RES treatment significantly reduced number of dead cells in both of the experimental groups (*P* < 0.01 and *P* < 0.001 respectively for ASC_EMS I_ and ASC_EMS II_). The same tendency was observed in β‐galactosidase quantification‐ increased senescence was observed in EMS group although AZA/RES treatment successfully inhibited accumulation of dye (Figure 3C). To support our stainings results, RT‐PCR for apoptosis‐related genes was performed. The apoptotic incidence in ASC_EMS_ was highly increased in comparison to control cells. However, co‐culture with AZA/RES significantly decreased the expression of p53 (Figure 3D), p21 (Figure 3E) and caspase‐9 (Figure 3F) indicating that it could inhibit ASC_EMS_ apoptosis. Moreover, cell cycle analysis revealed that AZA/RES reduced number of apoptotic cells in sub G1 phase (Figure 3G). Moreover, caspase‐3 amount in cells was visualized using western blot (Figure 3H). It decreased level was noted in both experimental groups. ELISA for p53 revealed increased amount of p53 in ASC_EMS_ in comparison to control cells (Figure 3I, *P* < 0.01). AZA/RES treatment decreased p53 amount in ASC_EMS I_ in comparison to ASC_EMS_ (*P* < 0.05).

### 3.4 AZA/RES treatment improved mitochondrial condition and reduced ROS accumulation in ASC_EMS_

**Figure 4:**
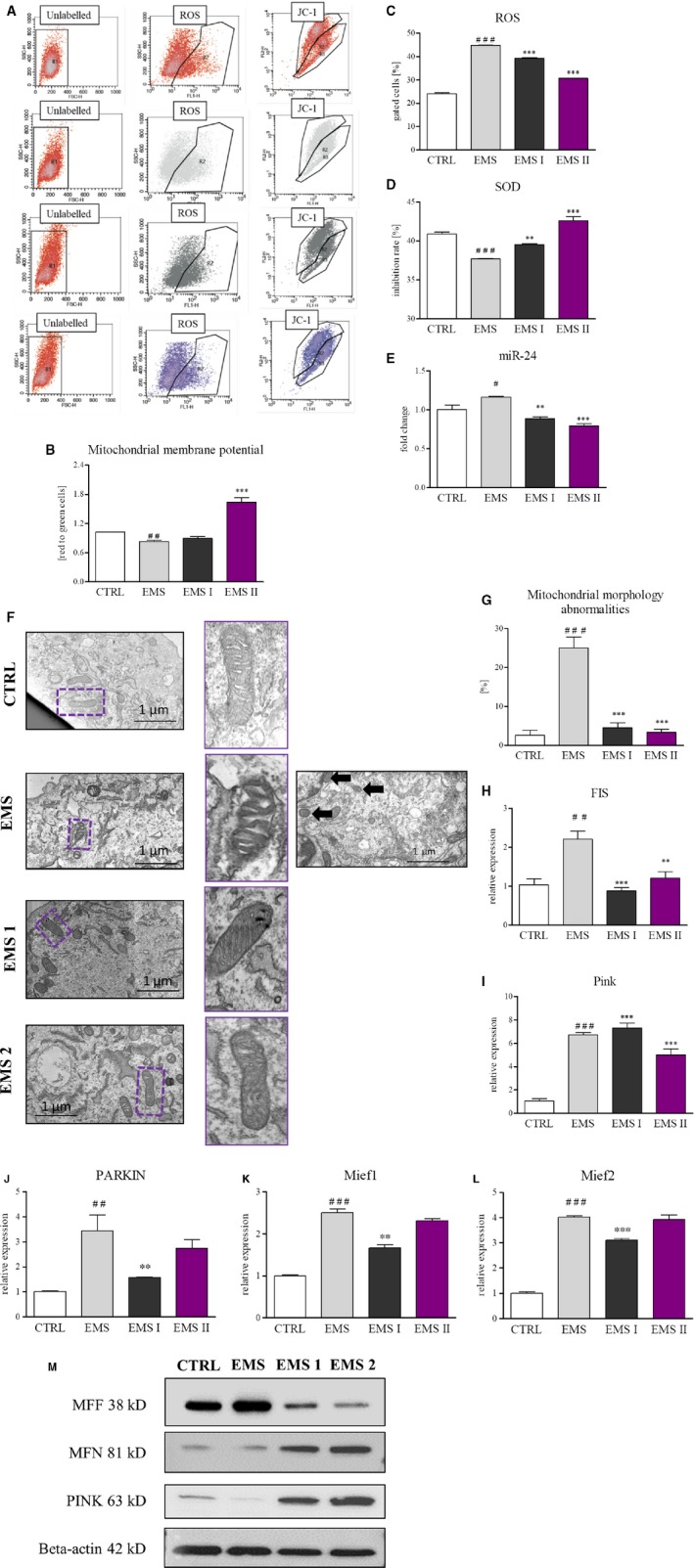
AZA/RES treatment improved mitochondrial condition and reduced ROS accumulation in ASC_EMS_. To investigate oxidative stress in cells, they were culture for 24 hours in control or experimental conditions and subjected to further analysis. MMP was measured using JC‐1 probe by flow cytometry (A). MMP depolarization in ASC_EMS_ was markedly reversed in ASC_EMS__II_ (B). Intracellular ROS level was stained with H2DCFDA and detected by flow cytometry. Results were respectively shown in the histograms (A). AZA/RES treatment significantly reduced ROS production in ASC_EMS_ (C). Antioxidative properties of cells were analyzed by spectrophotometric measurement of SOD activity (D). ASC_EMS__II_ displayed enhanced enzyme activity. Using RT‐PCR amount of miR‐24 was evaluated (E). TEM analysis showed ultrastructural changes in mitochondria morphology in ASC_EMS_ and it reversal after AZA/RES treatment (F). Boxed regions were magnified and showed on the left side. Based on representative photographs, quantification of aberrant mitochondria was performed (G). Mitochondrial fission was examined using RT‐PCR for FIS (H) while mitophagy was established by measuring the expression of PINK (I) and PARKIN (J). Moreover, we established that AZA/RES diminished expression of Mief1 (K) and Mief2 (L). Furthermore, using western blot amount of MFF, MNF, PINK were investigated (M). In treated cells, AZA/RES mitigated both, mitochondrial division and mitophagy. Scale bars: 1 μm. Results expressed as mean ± SD. Statistical significance indicated as asterisk (*) when comparing the result to ASC_CTRL_, and as hashtag (#) when comparing to ASC_EMS_. #*P* < 0.05; ##, **P < 0.01; ###, ***P < 0.001

Growing evidence indicates that impairment of mitochondria and accumulation of ROS contributes to aging of ASC limiting their therapeutic value. We therefore investigated wether AZA/RES cytoprotective effect was related to improvement of mitochondria in ASC_EMS_. In order to perform experiments, cells were seeded onto 24‐plates at the initial density 2 × 10^4^ and propagated in control and AZA/RES supplemented medium. To determine if AZA/RES impacted oxidative status and mitochondria condition of cells we measured accumulation of ROS, MMP, SOD activity, deeply visualized mitochondrial morphology using (SEM‐FIB) and assessed the expression of genes involved in regulation of mitochondrial dynamics. In order to perform the experiment, cells were treated with AZA/RES for 24 hours. As shown in Figure 4A, cells were subjected to flow cytometry analysis using JC‐1 probe (Figure 4A). Our results displayed that, MMP is significantly reduced in ASC_EMS_ in comparison to ASC_CTRL_. However, this alternation was markedly reversed after AZA/RES treatment, as indicated by the accumulation of JC‐1 aggregates (indicated as red to green ratio in Figure 4B). That phenomenon was only observed in ASC_EMS II_ which suggest, that higher concentration of RES are more beneficial in preventing mitochondrial damage. Interestingly, there was no significant change in those parameters mentioned above in ASC_EMS I_. MMP was slightly increased however without statistical significance in comparison to ASC_EMS_. To confirm improvement of mitochondrial functionality caused by AZA/RES, we investigated accumulation of intracellular ROS using H2DCFDA probe and flow cytometer (Figure 4A). Excessive ROS accumulation is a hallmark of mitochondria impairment. As shown in Figure 4C, number of ROS positive cells was greatly increased in ASC_EMS_ in comparison to healthy cells (*P* < 0.001). Treatment of cells with AZA/RES significantly diminished abnormal ROS accumulation in both experimental group (*P* < 0.001) although in ASC_EMS II_ more effectively which supports the thesis that higher RES concentration is required for better outcome of mitochondria improvement. After evaluation of oxidative stress, we decided to investigate whether AZA/RES simultaneously with decreasing ROS induce antioxidative response in cells. Antioxidative protection coming from SOD activity was ameliorated in ASC_EMS_, however AZA/RES treatment significantly restored its activity, especially in ASC_EMS II_ (Figure 4D). SOD activity in that group was greater than in control, untreated cells. MicroRNA (miRs) are small, about 22 nucleotides long, non‐coding RNAs able to inhibit mRNA translation or promote its degradation. Recent findings indicates on the role of miRs in differentiation, proliferation, apoptosis and modulation of mitochondrial metabolism. For that reason, we investigated the expression of miR‐24 in cells as its overexpression disrupts mitochondrial function. We discovered increased expression of miR‐24 in ASC_EMS_ in comparison to control cells, however AZA/RES treatment significantly reduced its expression in both experimental groups (Figure 4E, *P* < 0.01 and *P* < 0.001 respectively). To determine, whether AZA/RES influence morphology of mitochondria we performed TEM analysis. Analysis revealed, that ASC_EMS_ mitochondria were characterized by morphological aberrations like membrane raptures, vacuole formation and disarrayed cristae (Figure 4F). There were also mitochondria with rounded, swollen shape. In case of ASC_CTRL_, elongate, bean shape organelles were noted without any significant aberrations. Based on those parameters we quantified TEM photographs as shown in Figure 4G. Obtained results indicated that AZA/RES diminished mitochondrial damage as its significantly reduced abnormalities in mitochondrial morphology. Thus, we next investigated, if those changes results from mitochondrial dynamic shift. Mitochondrial fission is protective mechanism by which deteriorated organelles are arrested to ultimately be removed by the mitophagy process. Hence, we investigated the FIS expression using RT‐PCR. ASC_EMS_ were characterized by increased fission (Figure 4H) and increased expression of PINK (Figure 4I) and PARKIN (Figure 4J), both crucial to initiate mitophagy. Moreover, expression of Mief1 (Figure 4K) and Mief2 (Figure 4L) was diminished. Furthermore, amount of MFF, MNF and PINK were visualized by western blot (Figure 4M). We opined that enhanced mitophagy is protective mechanism which enables cells to maintain their stemness and compromise with excessive ROS as no mitophagy induction was noted in control cells. What is interesting, we noted reduced mitochondrial fission in experimental group supporting thesis about improvement of mitochondrial function. Especially because amount of MFN was increased in both experimental groups. Pink expression was upregulated in both experimental groups but ASC_EMS II_ more than in ASC_EMS I_. It may indicate that higher concentration of RES is necessary to rapidly remove and improve mitochondria condition.

### 3.5 AZA/RES treatment diminished formation of autophagosomes and autolysosomes in ASC_EMS_

**Figure 5:**
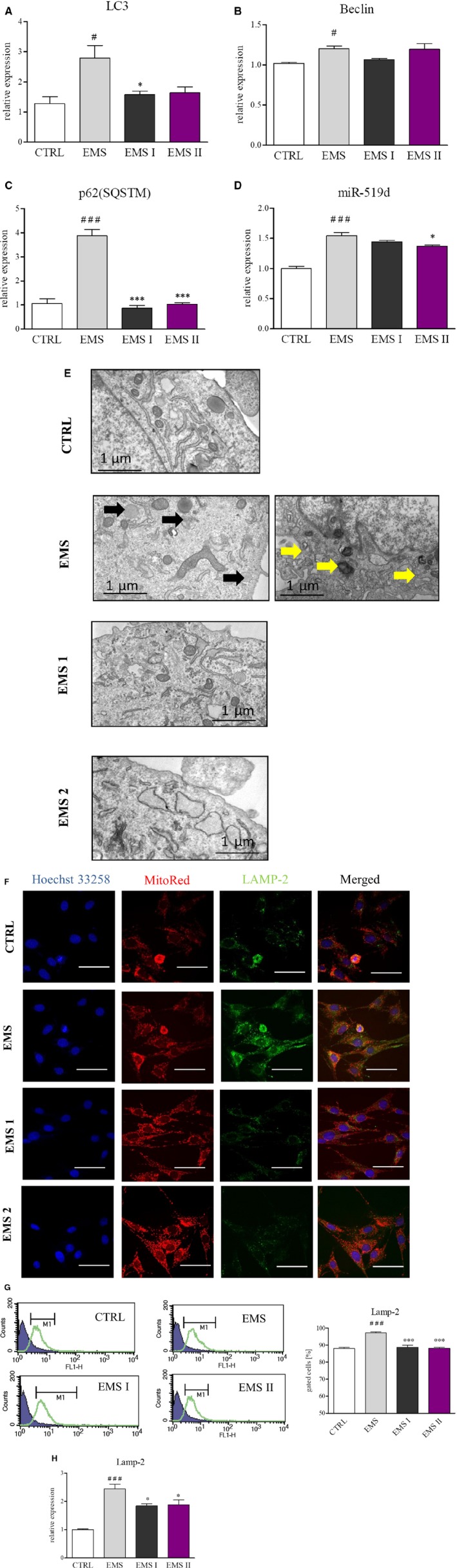
AZA/RES treatment diminished formation of autophagosomes and autolysosomes in ASC_EMS_. To investigate the autophagy in investigated cells, they were propagated in control or AZA/RES supplemented culture medium for 24 hours. Using RT‐PCR, expression of LC3 (A), Beclin (B), p62 (C) and miR‐519d (D) was evaluated. Formation of autophagosomes and autolysosomes was examined using TEM imaging (indicated with yellow arrows) (E). Obtained results displayed that autophagic flux in ASC_EMS_ was reversed resembling the autophagy level observed in ASC_CTRL_. Using double staining for mitochondria and anti‐LAMP2, we visualized mitophagy in investigated cells (F). Merged photographs indicated on enhanced mitochondria removal in ASC_EMS_ (F). In turn, mitophagy was partially mitigated after AZA/RES treatment. The percentage pf cells positive for LAMP2 was established by flow cytometry (G). Those result was supported by RT‐PCR for LAMP2 as well (H). Scale bars: 20 μm. Results expressed as mean ± SD. Statistical significance indicated as asterisk (*) when comparing the result to ASC_CTRL_, and as hashtag (#) when comparing to ASC_EMS_. #, *P < 0.05; ##, **P < 0.01; ###, ***P < 0.001

Our previous data strongly indicated, that enhanced mito‐ and autophagy are protective mechanism in ASC_EMS_. To extend our findings regarding autophagic shift in those cells, we investigated whether AZA/RES mediated restoration of ASC_EMS_ properties was in relation to autophagy modulation. Results from RT‐PCR showed reduction of LC3 (Figure 5A) in ASC_EMS I_ (*P* < 0.05). No significant differences in were found in the expression of Beclin in experimental group in relation to ASC_EMS_ (Figure 5B), however, marked reduction of p62 was noted (Figure 5C, *P* < 0.001). Expression of adipogenesis promoting miR519d was increased in ASC_EMS_, however we observed it decreased levels in ASC_EMS II_ (Figure 5D). TEM results also displayed that were few autophagosomes formation in control and both experimental groups (Figure 5E) while ASC_EMS_ were characterized by massive vacuoles and autophagosomes accumulation. ASC_EMS_ displayed increased mitophagy as shown in Figure 5G. Immunofluorescence analysis demonstrated that AZA/RES treatment significantly decreased the co‐localization of MitoRed labelled mitochondria and LAMP2‐positive lysosomes to that observed in control ASC from healthy individuals (Figure 5F). Meanwhile, flow cytometer results displayed that LAMP‐2 positive cell number was significantly reduced (*P* < 0.001) by AZA/RES treatment in both experimental group to similar level observed in ASC_CTRL_ (Figure 5G). Those observations were confirmed by RT‐PCR results, as LAMP2 mRNA level was diminished in those groups as well (Figure 5H).

### 3.6 AZA/RES decreased ER stress in ASC_EMS_

**Figure 6:**
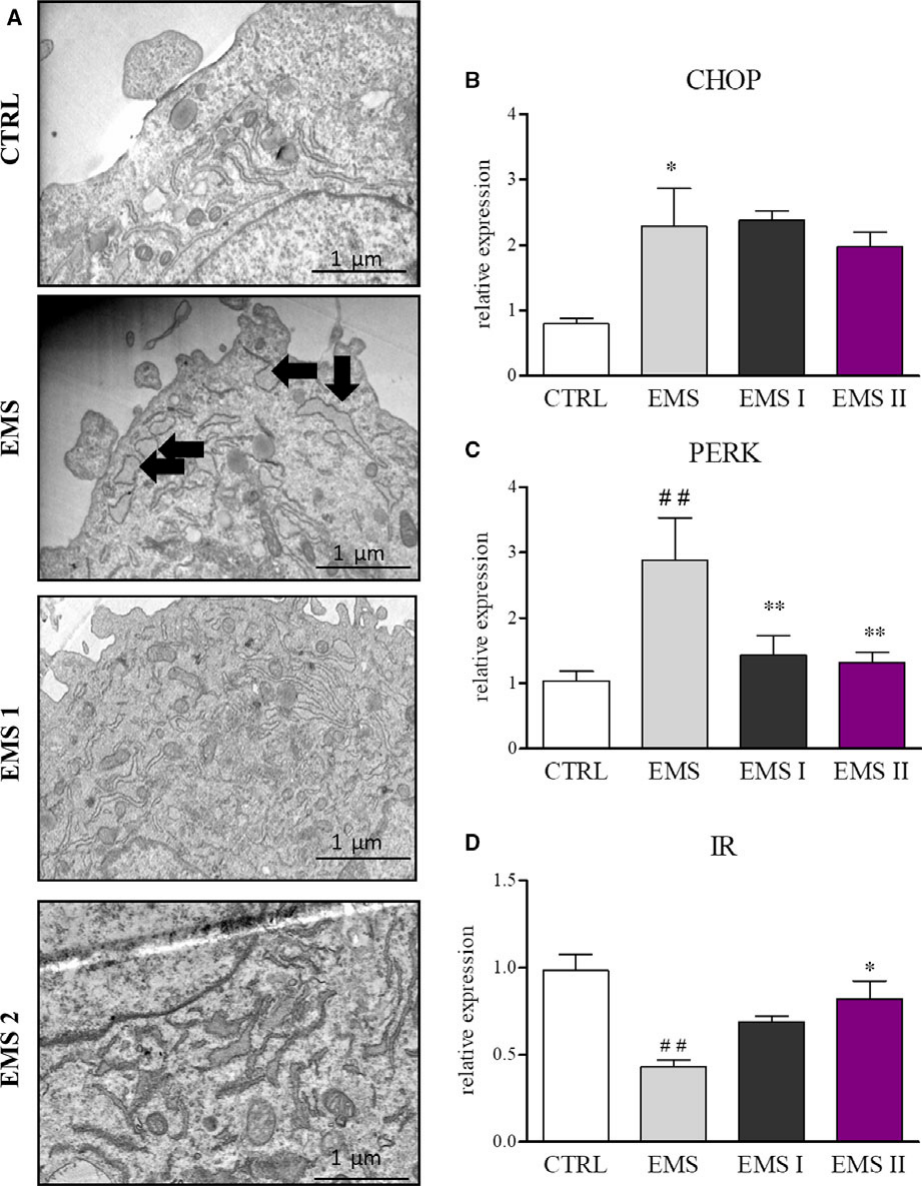
AZA/RES decreased ER stress in ASC_EMS_. In order to evaluate ER stress, cells were cultured for 24 hours in the control or AZA/RES supplemented medium. ER morphology was visualized using TEM (A) and obtained results revealed ER disruption in ASC_EMS_ (swollen, fragmented ER indicated with black arrows). In comparison to ASC_CTRL_, ASC_EMS_ were characterized by enhanced CHOP (B) and PERK (C) expression, nonetheless AZA/RES reduced PERK expression in ASC_EMS__II_. Moreover, AZA/RES treatment increased expression of IR (D) in ASC_EMS__II_. Obtained results indicated that AZA/RES capacity to reduce ER stress and augment IR expression. Scale bars: 400 nm. Results expressed as mean ± SD. Statistical significance indicated as asterisk (*) when comparing the result to ASC_CTRL_, and as hashtag (#) when comparing to ASC_EMS_. #, *P < 0.05; ##, **P < 0.01

Recently, it become more and more evident, that endoplasmic reticulum (ER) stress does affect and modify both, morphology and bioenergetics of mitochondria. It can induce mitochondria impairment by loss of MMP, fragmentation of mitochondrial network and mitophagy. Thus we investigated if AZA/RES influence ER stress in cells. TEM photographs demonstrated that, ER of ASC_EMS_ was characterized by enlarged lumen and swollen cisternae (Figure 6A). On the contrary, ASC_CTRL_ ER, was well developed, extended as a network that formed large ER sheets. Therefore we investigated using RT‐PCR mRNA levels of CHOP and PERK. CHOP expression was significantly up‐regulated ASC_EMS_ in comparison to control cells, although AZA/RES treatment did not significantly reduced its expression (Figure 6B). Similarly, PERK was also up‐regulated in ASC_EMS_, however AZA/RES markedly decreased its expression in experimental groups (Figure 6C). Furthermore, analysis of IR expression revealed its decreased transcript amount in ASC_EMS_ while AZA/RES increased its expression in ASC_EMS I_ (Figure 6D).

### 3.7 AZA/RES diminish apoptosis and autophagy in ASC EMS by the inhibition of mitochondrial fission

**Figure 7:**
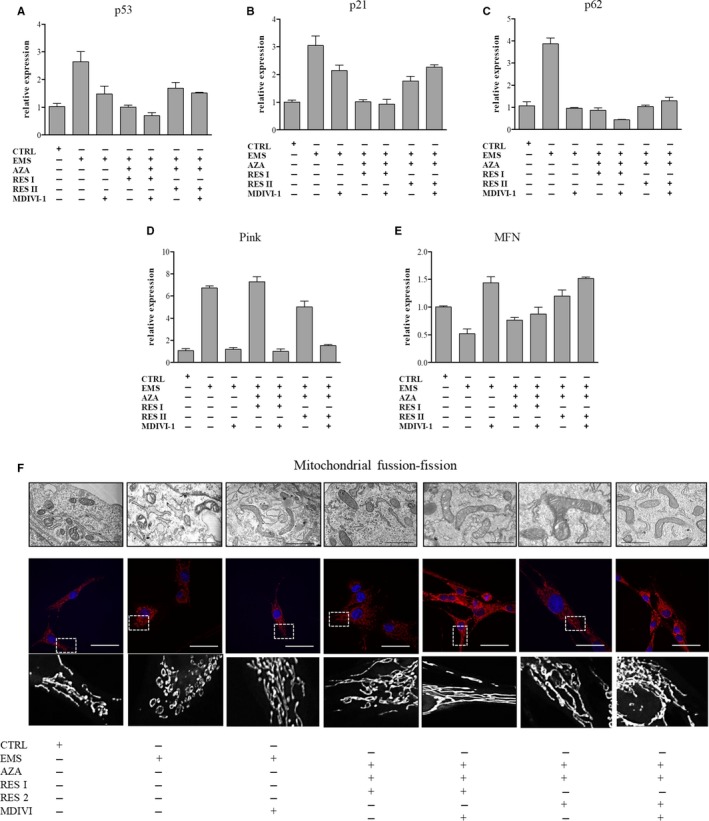
AZA/RES diminish apoptosis and autophagy in ASC_EMS_ by the inhibition of mitochondrial fission. In order to uncover the mechanism underlying AZA/RES beneficial effects on cells, they were cultured in control, AZA/RES and/or mitochondrial division inhibitor‐ MDIVI‐, supplemented medium. After 24 hours of propagation cells were subjected to RT‐PCR, TEM and confocal analysis. Using RT‐PCR expression of p53 (A), p21 (B), p62 (C), PINK (D) and MFN (E) was analyzed. Mitochondrial dynamics and morphology of mitochondrial net were further visualized using TEM and confocal microscope (MitoRed staining) (F). Boxed regions were enlarged and presented below MitoRed staining to visualize shape of mitochondrial network. Obtained results indicated that beneficial effects of AZA/RES result from inhibition of mitochondrial fission and modulation of autophagy in treated cells. Scale bars: TEM: 5 μm, confocal: 20 μm. Results expressed as mean ± SD. Statistical significance indicated as asterisk (*) when comparing the result to ASC_CTRL_, and as hashtag (#) when comparing to ASC_EMS_. #, *P < 0.05; ##, **P < 0.01; ###, ***P < 0.001

To further investigate the mechanism underlying the beneficial effects of AZA/RES on ASC_EMS_, we used mitochondrial division inhibitor‐ Mdivi‐1. In order to perform the experiments, cells were treated with AZA/RES at two different concentration and supplemented or not with the inhibitor for 24 hours. To evaluate its influence on cells, RT‐PCR,TEM and confocal analysis were performed. ASC_EMS_ were characterized by increased expression of p53 (Figure 7A) and p21 (Figure 7B) but this could be suppressed by AZA/RES and/or Mdivi‐1 treatment. Similar, the expression of p62 was brought back to levels observed in control cells after AZA/RES and/or Mdivi‐1 treatment (Figure 7C). However, AZA/RES do not promote fission and PINK expression (Figure 7D). It seems to be promoting mitochondrial fusion over fission as shown in Figure 7E. Deeper investigation on mitochondrial dynamics and it differences between groups was investigated with confocal and TEM microscope (Figure 7F). Obtained results indicated on increased fission in ASC_EMS_ in comparison to ASC_CTRL_. Treatment of ASC_EMS_ with Mdivi‐1 restored mitochondrial network strikingly resemble to one occurring in ASC_CTRL_. Similar observations were made in AZA/RES experimental groups, where mitochondria were long and elongated which supports our thesis that AZA/RES promotes fission while inhibits mitochondrial division.

### 3.8 AZA/RES treatment rejuvenates ASC_EMS_ through epigenetic alternations

**Figure 8:**
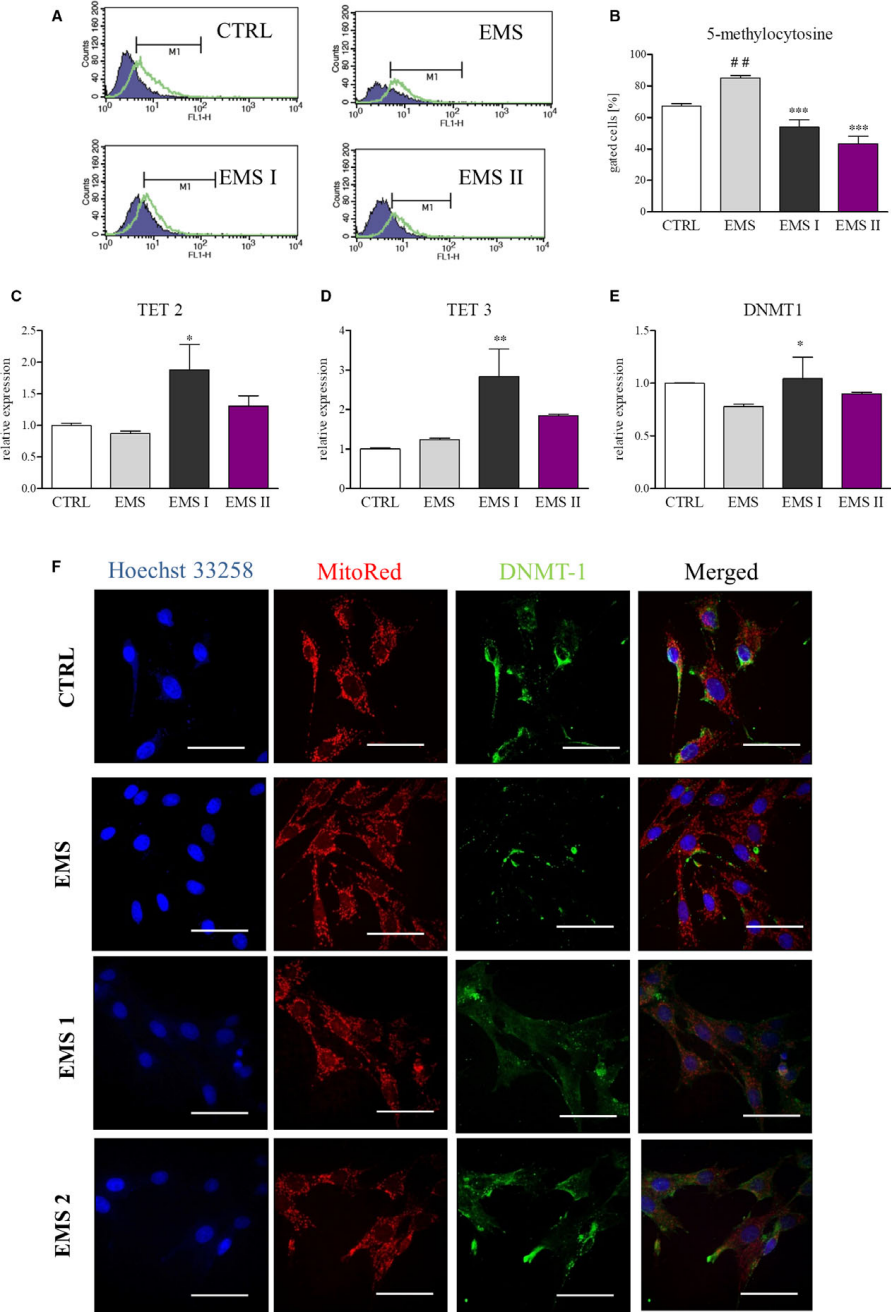
AZA/RES treatment rejuvenates ASC_EMS_ through epigenetic alternations. After 24 hours of culturing cells in control and AZA/RES supplemented medium, they were subjected to flow cytometry, immunofluorescence and RT‐PCR analysis. To evaluate levels of 5mC, cells were analyzed using flow cytometry (A). Obtained results indicated that AZA/RES significantly diminished DNA methylation in treated cells (B). Expression of TET 2 (C), TET 3 (D) and DNMT1 (E) was examined by RT‐PCR. Furthermore, localization of DNMT1 was assessed using immunofluorescence (F). Scale bars: 20 μm. Results expressed as mean ± SD. Statistical significance indicated as asterisk (*) when comparing the result to ASC_CTRL_, and as hashtag (#) when comparing to ASC_EMS_. *P < 0.05; ##, **P < 0.01; ***P < 0.001

In order to evaluate AZA/RES on epigenetic status of treated cells, cells were treated with AZA/RES for 24 hours and submitted for analysis. As AZA is well known inhibitor of DNA methylation, we investigated with flow cytometry the levels of 5‐methylocytosine (5‐mC) in DNA. ASC_EMS_ displayed increased methylation status in comparison to ASC_CTRL_, however AZA/RES treatment resulted in decreased levels of 5‐mC (Figure 8A). Furthermore, using RT‐PCR, expression of TET2 (Figure 8C) and TET3 (Figure 8D), both promoting demethylation. Although no differences were observed between ASC_CTRL_ and ASC_EMS_, ASC_EMS I_ were characterized by increased expression of both transcripts. No differences were noed in the expression of DNMT‐1 between investigated groups on mRNA levels (Figure 8E). Immunofluorescence staining for anti‐DNMT‐1 confirmed RT‐PCR results (Figure 8F).

## 4 DISCUSSION

In our previous studies, EMS has been recognized as a factor that significantly contributes to the deterioration of ASC functionality. Identification of mechanisms or agents that could reverse ASC impairment and enhance their therapeutic potential has become an urgent issue, as it was shown that allogeneic cells are not fully immune‐privileged, as previously thought. The results of this study have pointed out that AZA/RES combination can alleviate ASC_EMS_ deterioration and restore their physiological properties. Furthermore, we found that potential mechanisms underlying the cytoprotective effects of AZA/RES were related to modulation of mitochondrial dynamics and antioxidative action.

Our recent research has clearly indicated that age and disease strongly affect ASC physiological properties.^20^, ^42^ ASCs_EMS_ were characterized by the decreased proliferation rate, increased apoptosis and senescence, mitochondrial dysfunction as well as autophagic shift – a protective mechanism maintaining their stemness status. Therefore, alleviation of the negative impact of ageing is critical for therapeutic application of ASCs_EMS_. In this study, we demonstrated that AZA/RES can reverse physiological properties of these cells and attenuate their senescence.

The effects of RES on cellular senescence have been widely investigated, although the results are contradictory. Multiple studies, including our own, have pointed out that RES can attenuate cell ageing^31^, ^43^, ^44^; other have demonstrated that it can actually induce senescent phenotype, but in cancer cell lines.^45^, ^46^ However, the molecular mechanisms responsible for that activity were not fully elucidated. It is generally believed that a higher concentration (>25 μM) of RES causes apoptosis, while lower doses (<10 μM) exert anti‐ageing effects. In line with this evidence, we investigated the administration of two low concentrations (0.05 and 5 μM) of RES, and we found that both were able to attenuate the senescence of ASC_EMS_. In our earlier studies, we discovered beneficial effects of AZA on aged human ASCs. The results indicated that treating cells with AZA could be a justified intervention, capable of slowing down and even reversing age‐related changes in the investigated cells.^35^ Data presented by Yan et al^34^ supported our thesis, as these authors proved that AZA enhanced the osteogenic differentiation of aged ASCs by DNA demethylation. The beneficial concentration of AZA seems to be within the range of <5 μM. In the current study, based on our unpublished data, 0.5 μM of AZA worked more efficiently in combination with RES at two experimental concentration levels. We decided to combine those two agents in order to rejuvenate aged ASCs_EMS_, because ASCs_EMS_ are characterized by the accumulation of excessive ROS, mitochondria dysfunctions and increased DNA methylation.

The results have shown that AZA/RES treatment does not change cell identity. No differences in surface antigen expression were recorded after the treatment. For safe and effective therapy, it is crucial that cells retain their characteristics. Furthermore, it is strongly desirable to obtain a high number of cells in a short period of time, which not only advances the therapy, but also reduces its costs. Our current findings revealed that AZA/RES treatment markedly enhanced ASC_EMS_ proliferation, especially in ASCs_EMS II_ where the cell number was even higher than in ASCs_CTRL_. Moreover, it reduced the number of enlarged senescent cells in the culture, and at the same time increased the secretion of MVs and formation of cytoskeletal projections. These features are fundamental when considering the therapeutic application of cells, because enhanced secretory activity directly affects the regeneration process in vivo.

In this study, we discovered that AZA/RES significantly attenuated senescence and the number of dead cells in culture. In addition, it decreased the expression of apoptosis‐related genes, i.e. p53, p21 and caspase‐9. Reduction of apoptosis by AZA/RES was also proved by cell cycle analysis. Our results are consistent with the recent findings of Latorre et al^47^ who discovered that treatment with resveralogues reversed cellular senescence in human primary fibroblasts. Many other studies confirmed the beneficial effects of RES on MSC biology, mainly by enhancing their differentiation potential.^48^, ^49^, ^50^, ^51^

The antioxidant properties of RES are well documented, as it is known to protect both proteins and organelles from oxidation in time‐ and concentration‐dependent manner. Using oxidatively stressed erythrocytes, it has been shown that polyphenol exerts the best antioxidative protection between 30 and 60 minutes after addition.^52^ However, RES is not only a free radical scavenger by itself, but it can also modulate the activity of antioxidative enzymes. Studies have proved that RES can increase the amount of SOD, glutathione peroxidase and glutathione reductase.^53^ Our data confirmed that, because we observed significantly increased SOD activity in the experimental groups. Simultaneously, ROS levels were markedly reduced.

It should be noted that oxidative damage affects replication and transcription of mitochondrial DNA, resulting in the impairment of mitochondrial functionality and quality. Both mitochondria dysfunction and excessive ROS accumulation are frequently observed in diabetes, and thus the MS plays an important role in mammalian ageing. Since ASCs_EMS_ are characterized by mitochondrial deterioration, including morphological abnormalities, decreased MMP and increased mitophagy, we investigated whether AZA/RES is able to reverse the pathological state of these organelles. In the present study, we discovered that AZA/RES induced the attenuation of mitochondrial oxidative stress and the impairment of ASCs_EMS_. Our observation strongly suggests, that the protection of mitochondria by RES can be attributed to its anti‐ageing action and is consistent with previously published studies.^54^ Importantly, AZA/RES treatment improved mitochondria status in ASCs_EMS_ to the levels observed in healthy control cells; what is more, Pink and Parkin expression was reduced indicating mitophagy inhibition. Moreover, regulation of oxidative stress by AZA/RES may be mediated via miR‐24, because miR‐24 expression was significantly reduced in treated cells.

Autophagy is a process by which cytoplasmic components are sequestered in autophagosomes and then transferred into lysosome for degradation. Increasing evidence suggests that augmented autophagy is an effective mechanism inhibiting apoptosis in many diseases.^52^ However, the role of autophagy in stem cells has only been studied in several recent works. The results presented by Ho et al^55^ have demonstrated that autophagy with age becomes a key mechanism preserving the regenerative capacity of old haematopoietic stem cells. It was also shown that autophagy inhibition in adult stem cells abolished their self‐renewal capacities and differentiation potential.^56^ Our own data have revealed that the “autophagic shift” in ASCs_EMS_ is a protective mechanism helping cells to overcome oxidative damage and remove impaired mitochondria. Furthermore, this mechanism supplies precursors for macromolecule synthesis, thereby allowing cells to maintain their stemness status and ability to differentiate into multiple lineages.^22^, ^23^ In the present research, AZA/RES treatment of ASCs_EMS_ effectively rejuvenated these cells, so that autophagy could be down‐regulated to the basal level observed in ASCs_CTRL_. The expression of autophagy‐related genes, including Beclin, p62 and LAMP2 was reduced after treatment. We have been suggested that AZA/RES improved the metabolic status of ASCs_EMS_, so that no compensative mechanism was required any longer. Our results contributed to the discrepancy related to the RES mechanism of action. While previous evidence indicated that RES treatment induced autophagy, we observed that AZA/RES treatment markedly inhibited the autophagic response. A similar RES characteristic was observed in the study conducted by Armour et al^57^ who discovered that RES attenuated autophagy in a number of cell lines in response to nutrient limitation or through a pathway independent of the known target, SIRT1. These effects can be mediated by modulation of miR‐519a activity, which can directly down‐regulate Beclin‐1, thereby decreased autophagy. Those data are consistent with our results, as we observed up‐regulation of miR‐514a in ASCs_EMS_, where the autophagic shift was recorded and it decreased after AZA/RES treatment. The obtained results indicate that beneficial effects of RES do not result from autophagy induction, but rather from autophagy inhibition. It is tempting to speculate that RES maintains cellular homeostasis through dual regulation under different conditions.

ER stress is markedly associated with oxidative stress and mitochondrial dysfunction. There is an accumulating body of evidence indicating that prolonged ER stress is involved in the development of many metabolic disorders, including diabetes and liver disease.^58^ Our results strongly support that thesis, as ASCs_EMS_ displayed disturbances in ER morphology, including swelling and fragmentation. Furthermore, RT‐PCR analysis revealed significantly increased expression of two stress genes related to ER: CHOP and PERK. Thus, therapeutic interventions targeting ER stress response would be potentially valuable in the treatment of diseases associated with prolonged ER stress. Our data revealed that AZA/RES treatment diminished ER stress in ASCs_EMS_ by significantly down‐regulating PERK expression. Our data are in line with Gaballah et al,^59^ who observed that RES exerted modulatory effects on ER stress‐associated apoptosis in rats with rotenone‐induced Parkinson's disease. However, there are conflicting data implicating that RES can triggered ER stress^60^, ^61^ However, increased ER stress caused by RES occurred mostly in cancer cell lines and was strictly correlated with polyphenol concentration and time of supplementation. We investigated whether disturbances in IR and FOXO‐1 expression occurred in ASCs_EMS_, because ER appeared to act directly as a negative regulator of insulin signalling pathway.^62^ We have found that IR expression is significantly down‐regulated in ASCs_EMS_, which may indicate that IR in EMS horses affects not only insulin‐sensitive tissues, but also adipose‐derived stem cells. However, the mechanism of IR in stem cells remains elusive and clearly needs to be further investigated. It should be noted that AZA/RES significantly increased IR expression in ASCs_EMS II_, which is consistent with the experiments conducted by other researchers, who found that RES considerably improved insulin sensitivity and glucose control in subjects with diabetes.^63^

Mitochondrial morphology is mediated by the balance between fission and fusion processes. Fission activation occurs in response to excessive ROS and induces mitochondrial impairment. FIS activation in ASCs_EMS_, is evoked through increased oxidative stress and mitochondrial damage, leading to mitochondrial net fragmentation. However, AZA/RES preserved MFN expression and inhibited FIS activation, thereby protecting mitochondrial integrity and functionality. AZA/RES protected mitochondria from fission and, in consequence, diminished ASC_EMS_ apoptosis. Treatment with AZA/RES reduced the number of senescent cells with fragmented mitochondria and promoted increased fusion over fission events. Cells treated with AZA/RES exhibited long and connected mitochondrial net. These results strongly suggest that RES does not only act as an antioxidative agent, as it rather has the ability to modulate mitochondrial dynamics to maintain their proper function. However, more research is required to clarify mechanisms underlying those effects.

We have also verified how AZA/RES supplementation affects the epigenetic status of the investigated cells. AZA/RES treatment markedly reduced methylation status of ASCs_EMS_ and simultaneously increased the expression of TET2 and 3 other genes. It agrees with our previous data regarding the effects of AZA on aged human MSCs, where its beneficial effect on these cells was proved.^35^ A study conducted by Rui et al revealed that epigenetic alternations of MSCs could be an attractive way to control their differentiation.^64^ Epigenetic regulation, including DNA methylation and histone modification plays a key role in the process of differentiation. Therefore, we have been suggested that AZA/RES‐treated cell would display increased differentiation potential. Similar results were presented by Yan et al^34^ who observed that AZA treatment of aged ASCs improved their osteogenic differentiation potential and induced TET family gene expressions.

In summary, we have demonstrated that AZA/RES treatment of ASCs_EMS_ is able to rejuvenate these cells by modulating mitochondrial dynamics, in particular by promoting mitochondrial fusion over fission. Our findings offer a novel approach and potential targets for the beneficial effects of AZA/RES in ameliorating stem cell dysfunctions. Supplementation of culture media with AZA/RES, prior to clinical application of cells, seems to be fully justified, since these rejuvenated cells may exert markedly improved therapeutic potential in vivo. However, additional research is needed to further evaluate the potential benefits of such therapy in horses.

## CONFLICT OF INTEREST

The authors confirm that there are no conflicts of interest.
